# Pneumomediastinum and Subcutaneous Emphysema After Rapid Mediastinal Mass Regression

**DOI:** 10.1002/jha2.70397

**Published:** 2026-09-23

**Authors:** Fahad Alsayed, Ailish Coblentz, Oussama Abla

**Affiliations:** ^1^ Division of Haematology/Oncology The Hospital for Sick Children University of Toronto Toronto Ontario Canada; ^2^ Department of Diagnostic Imaging The Hospital for Sick Children University of Toronto Toronto Ontario Canada

1

A 16‐year‐old boy presented with right cervical lymphadenopathy, facial redness, venous distention in the supine position, dizziness, shoulder pain and chest wall telangiectatic changes compatible with superior vena cava syndrome. Chest computed tomography (CT) showed a bulky anterior mediastinal mass compressing the airway and great vessels, with pleural and pericardial effusions (Figure 1a). Blood work showed hyperuricemia and hyperleukocytosis, with peripheral blood flow cytometry showing T‐lymphoblasts. He was therefore diagnosed with T‐cell acute lymphoblastic leukaemia, with no central nervous system or testicular involvement. He received a four‐drug induction chemotherapy regimen consisting of dexamethasone, vincristine, daunorubicin and asparaginase with intrathecal methotrexate.

**FIGURE 1:**
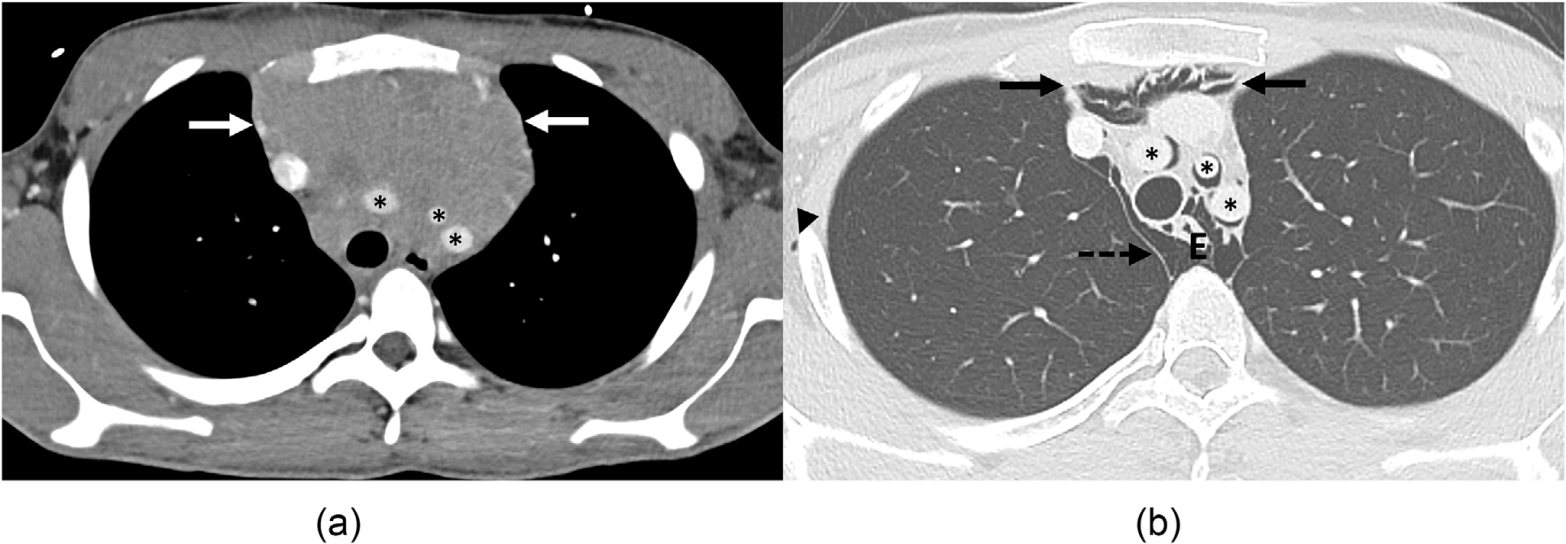
(A) Axial CT image (soft tissue window) obtained at time of diagnosis shows a large solid mass centred in the anterior mediastinum (white arrows) with encasement of the major arch vessels (*). (B) Follow‐up axial CT (lung window) obtained 1 month later depicts resolution of the mediastinal mass with new widespread pneumomediastinum, within the anterior mediastinum (black arrows), surrounding the arch vessels (*), encasing the oesophagus (E), throughout the posterior mediastinum (dashed arrow) and dissecting into the right lateral chest wall (arrowhead).

At the end of induction, chest CT showed that the mediastinal mass had nearly resolved (Figure 1b), with re‐expansion of the airway and great vessels and resolution of the effusions. Unexpectedly, there was extensive pneumomediastinum dissecting into the soft tissues of the neck, both axillae and the chest wall (Figure 2). He reported only sensation of pressure and discomfort in the right ear when sitting upright, and had a palpable cervical crepitus; he was otherwise afebrile and had no respiratory symptoms or chest pain. There was no pneumothorax, and no preceding central venous catheter, instrumentation or positive‐pressure ventilation. He was managed conservatively, chemotherapy continued uninterrupted, and a follow‐up chest x‐ray showed that the leak had resolved. He is now at almost 3 months from diagnosis without any symptoms related to pneumomediastinum.

**FIGURE 2:**
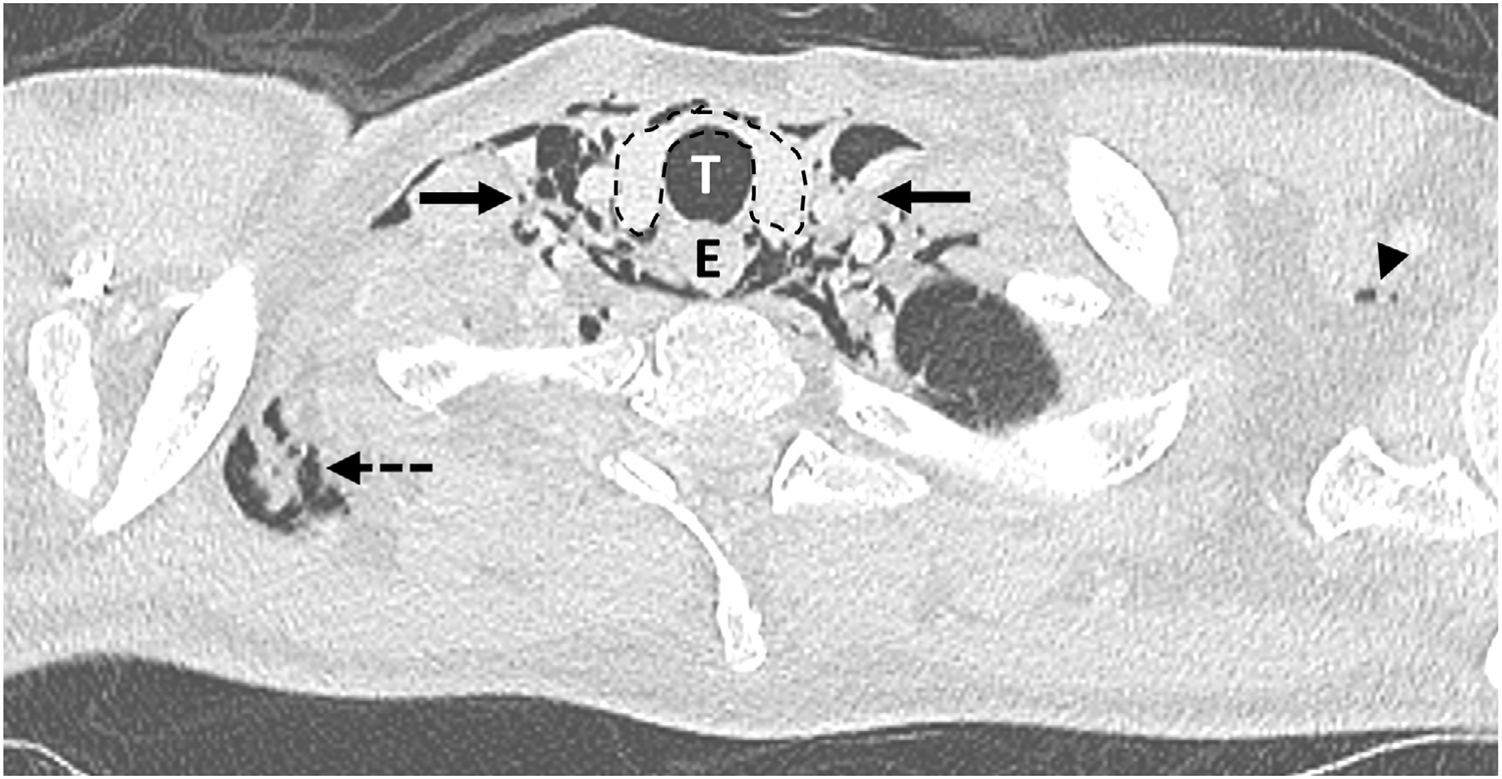
Axial CT (lung window) at the level of the inferior neck shows upward dissection of pneumomediastinum into: the soft tissues of the anterior neck (black arrows), surrounding the thyroid gland (hashed outline), trachea (T), oesophagus (E), the right posterior triangle (hashed arrow) and left axilla (arrowhead).

Pneumomediastinum is a rare complication of bulky mediastinal lymphoid malignancies, described after treatment for mediastinal large B‐cell lymphoma and other chemosensitive tumours [1, 2, 3, 4]. The likely mechanism is the Macklin effect [5], consisting of alveolar rupture with air tracking along bronchovascular sheaths into the mediastinum. Rapid chemotherapy‐induced tumour regression, with air escaping into the involuting mass, is the likely cause; raised intrathoracic pressure may also contribute [6, 7]. Presentation can be subtle: instead of chest pain or dyspnoea, the only sign may be neck pressure with cervical crepitus, as in our case, or it may be found incidentally on repeat chest imaging [6].

In a neutropenic child treated with chemotherapy, new mediastinal air should not be assumed to be benign. In this case, the lung parenchyma showed no consolidation, cavitation or suspicious nodules, with no oesophageal thickening or airway mural defects, making necrotising infection (*Pneumocystis jirovecii*, invasive fungal disease, non‐tuberculous mycobacteria) and aerodigestive perforation unlikely [8, 9]. Once these are excluded, most cases resolve on their own. Observation, analgesia and supplemental oxygen can be sufficient, and intervention is only needed for tension physiology, pneumothorax or airway compromise [7, 10]. A consultation with a respirology specialist is recommended. Finally, there is no need to interrupt chemotherapy.

## Author Contributions

F.A. conceived the report and drafted the manuscript. A.C. selected and interpreted the radiological images. O.A. supervised clinical management and critically revised the manuscript. All authors approved the final version.

## Funding

The authors have nothing to report.

## Consent

Written informed consent for publication was obtained from the patient's parent/legal guardian, together with the patient's assent. All identifying information has been removed from the images.

## Conflicts of Interest

The authors declare no conflicts of interest.

## Data Availability

Data sharing is not applicable to this article as no datasets were generated or analysed during the current study.
